# Virtual twin for healthcare management

**DOI:** 10.3389/fdgth.2023.1246659

**Published:** 2023-09-15

**Authors:** Thomas M. Polasek

**Affiliations:** ^1^ Certara, Princeton, NJ, United States; ^2^ Centre for Medicines Use and Safety, Monash University, Melbourne, VIC, Australia

**Keywords:** virtual twin, precision medicine, personalized medicine, precision dosing, pharmacogenomics (PGx)

## Abstract

Healthcare is increasingly fragmented, resulting in escalating costs, patient dissatisfaction, and sometimes adverse clinical outcomes. Strategies to decrease healthcare fragmentation are therefore attractive from payer and patient perspectives. In this commentary, a patient-centered smart phone application called Virtual Twin for Healthcare Management (VTHM) is proposed, including its organizational layout, basic functionality, and potential clinical applications. The platform features a virtual twin hub that displays the body and its health data. This is a physiologically based human model that is “virtualized” for the patient based on their unique genetic, molecular, physiological, and disease characteristics. The spokes of the system are a full service and interoperable electronic-health record, accessible to healthcare providers with permission on any device with internet access. Theoretical case studies based on real scenarios are presented to show how VTHM could potentially improve patient care and clinical efficiency. Challenges that must be overcome to turn VTHM into reality are also briefly outlined. Notably, the VTHM platform is designed to operationalize current and future precision medicine initiatives, such as access to molecular diagnostic results, pharmacogenomics-guided prescribing, and model-informed precision dosing.

## Healthcare fragmentation—a problem for patients and payers

Healthcare is increasingly fragmented. Patients, particularly those with chronic diseases, navigate complex healthcare systems with many service providers but often no-one “captaining the ship”. Payers mainly finance discrete services and episodes of care rather than wholistic care and clinical outcomes. The cost of healthcare continues to increase in real terms and as a proportion of gross domestic product (GDP) in Western countries (1).

There are many reasons for healthcare fragmentation. Some of these include the following. First, medical generalists, including family physicians in the United States (US) and general practitioners (GPs) elsewhere, have been devalued to a medical triage role in many well-serviced and high-resourced locations e.g., metropolitan centres. This is encouraged by governments, the medical hierarchy, and medical indemnity providers, but, ultimately, increases the number of high paid medical specialists. Second, evidence-based medicine demands specialist care if available. Indeed, patients with multiple comorbidities attend numerous medical specialists who apply clinical practice guidelines (CPGs) within their own speciality, sometimes to the determent of overall care. An example is the prescribing of a set list of drugs after myocardial infarction without consideration of potential interactions with existing drugs for pre-existing comorbidities, resulting in drug-drug interactions and adverse effects (2, 3). Third, allied health providers are working in areas traditionally serviced by family doctors, such as chronic disease management e.g., pharmacist and nurse prescribing from restricted drug formularies (4, 5). Fourth, specialized novel services that are expensive are increasingly added to healthcare budgets e.g., genetic testing (6). Finally, there are sometimes unrealistic societal expectations to provide treatments for all, independent of disease trajectory, age, and social circumstances (7) e.g., major surgery for small bowel obstruction in a patient with advanced dementia and very short life expectancy.

Given the decentralized models of patient care and the unsustainable costs of healthcare (>10% of GDP in Western countries), strategies to reduce healthcare fragmentation are attractive from payer and patient perspectives (8). This commentary introduces Virtual Twin for Healthcare Management (VTHM), a conceptual patient-centred smart phone application that disrupts the healthcare sector by addressing fragmentation and transferring control of health information from multiple sources to the patient. The system is a combined personal- and electronic-health record (PHR/EHR). Importantly, VTHM is designed to deliver precision medicine initiatives as they roll-out in more affluent economies, such as access to molecular diagnostic results, pharmacogenomic (PGx)-guided prescribing, and model-informed precision dosing (MIPD) (9–11).

## Current e-health systems

During their interactions with healthcare, patients tell their story repeatedly, relating medical history, allergies, current and past medications etc., time and time again to multiple people. Repetition is often exhaustive for patients with multiple chronic medical conditions and for their families and carers. Electronic-health records (EHRs) have been implemented to better capture this information, with the aims of improving continuity of care, patient safety and patient satisfaction (12). However, such e-health systems are often locally oriented, rarely communicate well with each other, and are difficult to access, either by healthcare providers without access, or by patients themselves.

Although some e-health systems are superior at linking patient data between various EHRs, for example Surescripts® is a US e-prescribing platform that covers about 90% of prescriptions in the community setting, there is considerable redundancy in this area. Information technology providers promise novel solutions but deliver little innovation to improve functionality, workflow and, critically, interoperability. Basic information that is essential for medical care, such as the patient's medical history, is collected repeatedly and stored in numerous e-health systems, whilst detailed information, such as a complete medical assessment with treatment plan from an experienced medical specialist, remains buried in a specific system because of access restrictions. This leads to expensive service repetition. A common example is the re-ordering of blood tests by different healthcare providers based on the pathology service they use.

## Virtual twin for healthcare management

The only common factor linking all the fragments of healthcare together—i.e., multiple healthcare providers, multiple healthcare records (paper, personal, electronic), multiple medication management systems, multiple pathology services, multiple medical imaging services etc.—is the patient. To date, attempts to centralize health information with national EHRs have had limited success. For example, the Australian Government started “My Health Record” in 2010, but participation by healthcare providers is relatively low and patients are resistant over privacy issues (13, 14). Personal health records have also been implemented, but again, these are locally oriented and focused on single chronic diseases, such as diabetes and heart failure, rather than providing more universal platforms for healthcare management (15).

**Figure 1:**
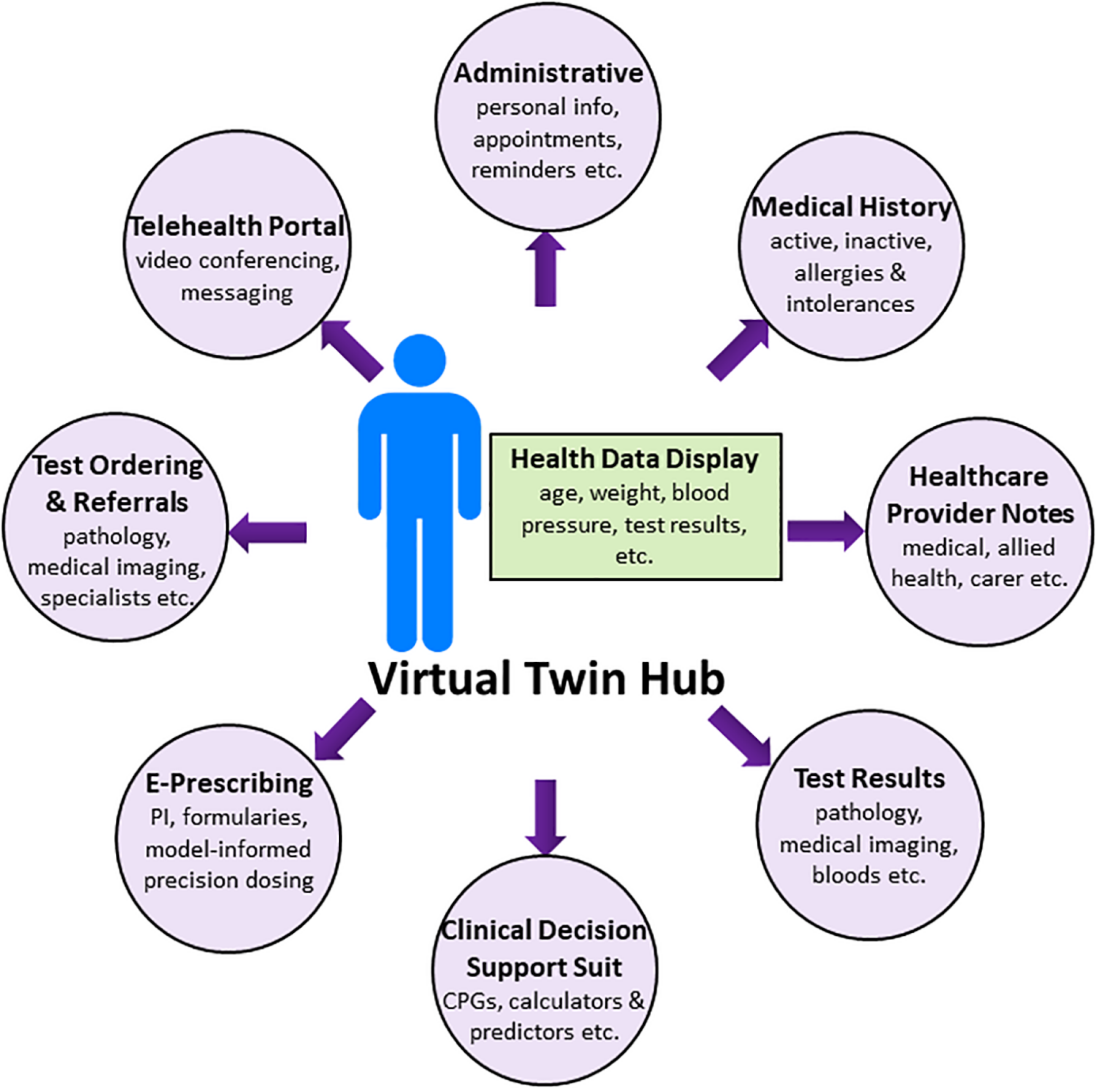
Organizational layout of virtual twin for healthcare management.

With the advent of ubiquitous smart phone use, this device is the ideal platform for patient control of their health information. Virtual Twin for Healthcare Management (VTHM) is a multi-functional patient-centred smart phone application using a human model at the hub (“virtual twin”—see below) and EHR modules at the spokes. The application is in the design and proof-of-concept phases but is presented here showing how VTHM would ideally operate in the future. It is important not to confuse the aspirational nature of VTHM with current actual capabilities. Indeed, this perspective describes the “finished product”, and many challenges must be overcome on the path from concept to reality (see later section). With this in mind, the organizational layout of VTHM is shown in the Figure 1. Data is stored on a secure server, allowing healthcare providers to access the VTs (virtual twins) of their patients from any device with an internet connection.

## What is a virtual twin?

A VT is a physiologically based (PB) human model constructed from a base model that is “virtualized” with each individual's unique genetic, molecular, physiological, and disease characteristics (16). It is the hub of VTHM and allows the user to visualise the body and its health data. An avatar of the patient overlays organ systems (“systems data”) arranged and separated with bloods flows, and tissue perfusion is modelled with differential equations based on physiology. The software that powers VT is Simcyp (Certara, USA), which is used to simulate drug pharmacokinetics (±pharmacodynamics) in the pharmaceutical industry (17, 18) and is increasingly being studied as a MIPD tool in clinical practice (19–22).

The systems data required to make a VT comes from sources that already inform medical decision making, including the prescribing of drugs. Data include basic demographics (age, weight, height, sex, ethnicity), pathology results (liver function tests, creatinine clearance, inflammatory markers etc.), biomarkers of disease and therefore potential treatment monitoring (real-time monitoring of blood glucose, prostate specific antigen, etc.), medical imaging (liver size, kidney size, ECHO cardiac output data etc.), and any values for physiological/molecular structure or function considered necessary for disease and treatment modelling and simulation. A Monte Carlo approach is employed to generate realistic values for unknown parameters of the system based on population covariate relationships whenever actual values are unknown e.g., the liver size of a Japanese male aged 50 years or the glomerular filtration rate of a 90 yo Caucasian female weighing 45 kg (17).

## Virtual twin is a dynamic physiologically based human model

Virtual twin is a dynamic PB model that changes as the patient ages (23). The VT also reflects medical changes. When a patient is diagnosed with a new disease, the pathophysiology changes that occur in the condition, if known, are updated in VT (24). When a woman becomes pregnant, the physiological system changes that occur during pregnancy are automatically activated (25). When information is known about drug metabolizing enzyme and transporter abundances and/or activities (26, 27), or a patient takes a PGx test to determine the genotype of a drug metabolizing cytochrome P450 enzyme (CYP) (28), this information is added to their VT to increase the level of individualisation (referred to as “virtualisation”) (20). When drug changes are made, drug-drug interactions, drug-gene interactions and other variables are considered as VT uses MIPD (see below) to predict drug pharmacokinetics (±pharmacodynamics).

## Functions of virtual twin for healthcare management

### Health data display

As described above, the VT hub is the patient's avatar that displays health data updated in real time. For example, age, weight, blood pressure, drug adherence, blood glucose concentrations, precision dosing targets (e.g., drug concentrations from therapeutic drug monitoring) (29) and key pathology findings, such as erythrocyte sedimentation rate (ESR), can be flagged for display. Lifestyle data from fitness and diet applications is incorporated when available. Displayed data are bespoke for each user and managed by each user so that only relevant information is presented.

### Full service electronic-healthcare record

The spokes of VTHM are the modules of an EHR, where patient demographics, appointment schedules, healthcare provider notes, pathology and medical imaging results, medical and medication histories, including information on therapeutic outcomes, drug preferences and allergies, and other healthcare information is written, stored, and accessed by those with permission (Figure 1). Requests for drugs (e-prescribing—see below), pathology (blood tests, medical imaging), referrals and other services are made via the spokes. In short, VTHM is a full service and interoperable EHR that transfers data to and from established major commercial EHRs.

### Clinical decision support system (CDSS)

The VTHM provides a broad range of active and passive clinical decision support tools, with the suit and functionality available to each user tailored to their role (15). In this way, different doctors have access to different tools applicable to their clinical practice. Likewise for nursing staff and the various allied health professions such as clinical pharmacy (30). Patients are less likely to use these functions, but they are available to them if desired. Examples of clinical decision support tools include, but are not limited to, the following:

i) Diagnostic decision support.
ii) Links to international, national and/or local CPGs e.g., UpToDate®, National Comprehensive Cancer Network [NCCN Guidelines®], LactMed® etc.
iii) Calculators and predictive algorithms e.g., renal function calculators (eGFR), CHADsVASC scores to predict the rates of ischaemic stroke in patients with atrial fibrillation, pharmacogenomics benefit score (PGxBS) to quantify the congruency between PGx test results and medication-related problems (31) etc.
iv) e-Prescribing, including customized drug order sets (e.g., anaesthetics), drug-drug interaction and allergy checking and alerts, drug safety monitoring (e.g., timing of blood glucose measurements), and precision dosing (see below).
v) Follow-up and treatment reminders to improve adherence.

#### Precision dosing tool

Part of the e-prescribing CDSS in the EHR allows for “precision dosing”—the tailoring of drug doses based on the patient's individual characteristics (32). Following drug selection, which is determined by diagnosis, and guided by CPGs, cost, and physician and patient choice, advice on dose selection is given via multiple sources (32):

i) Product information (PI) and drug monographs in commercial or independent drug information resources.
ii) Empirical dosing in which biomarkers from ongoing drug therapy are used to guide future dose selection e.g., International Normalized Ratio for warfarin.
iii) Pharmacogenomic testing to determine whether the patient has the correct molecular target (pharmacodynamics) (33), is at increased risk of extreme high or low drug exposure (pharmacokinetics) (34), or has an immune system genetic variant associated with severe adverse drug reactions (35).
iv) Model-informed precision dosing. Various types of models are used to individualized doses for narrow therapeutic index drugs in difficult to dose patients. Basic algorithms, population pharmacokinetic/pharmacodynamics (PK/PD), full or partial physiologically based pharmacokinetics (PBPK), combined approaches, or models linked to therapeutic drug monitoring of drug concentrations, are all examples of MIPD approaches that have been successfully applied in clinical practice (11, 36–38). Since VT is a PB human model, key input data for MIPD is readily available to support all modeling approaches e.g., age, sex, weight, CYP polymorphisms etc. (11).

Importantly, VT can provide advice on drug and dose selection across all points of contact with healthcare systems, from acute presentations in emergency departments to regular check-ups with family doctors for chronic health problems. Vancomycin dosing for a VRE positive infection in a child being treated in paediatric intensive care is an example of an acute need for precision dosing (39). Recommendations in general practice about the best additional anti-diabetic drug to start for a woman with type 2 diabetes mellitus who is now pregnant is an example of MIPD use in the chronic setting (40).

#### Telehealth

This part of VTHM is used for telehealth appointments and additional communications between patients and healthcare providers. For example, patients can set their priorities for upcoming appointments via secure messaging to a provider *a prior*, thus allowing them to efficiently address the patient's concerns i.e., improved patient-provider alignment (41). Follow-up quick communications also occur, thus reducing the costs of unnecessary appointments e.g., resolution of a symptom or problem, establishing a new treatment goal etc. A messaging system for secure between provider communication is available for confidential discussions.

## Case studies

A series of theoretical case studies based on real scenarios are presented here to show how VTHM could potentially improve patient care and clinical efficiency.

### Case 1—acute hospital admission

An elderly man with multiple comorbidities is traveling interstate with his son to watch his favourite football team. During the flight he develops chest pain and is rushed to an emergency department after landing. A thorough medical history is available at his home hospital, which includes multiple drug allergies and previous severe adverse drug reactions. During the hospital admission, for which he requires coronary revascularisation, the gentleman and his son are unable to recall the allergy/intolerances to medications, and he is given clopidogrel, the perpetrator of a previous gastrointestinal haemorrhage, potentially caused by his “rapid metabolism” CYP2C19*17 genotype (42, 43). This could be avoided if the gentlemen pulled out his mobile phone, giving the emergency and coronary care doctors access to his full medical record instantly.

### Case 2—integrated chronic disease management

An overweight businessman with a cardiovascular risk score of >10% in the next 5 years is provided with lifestyle advice and tips from VTHM, together with a continuous feedback control loop for his blood glucose monitoring to enable precision dosing of insulin (44). Information from fitness, diet and fasting smart phone applications are integrated into VTHM to monitor progress towards weight loss goals. The gentleman finds this approach motivating and reviews progress charts with his GP regularly.

### Case 3—rare disease patient support

A middle-aged man with undiagnosed myopathy has a new genetic test result that could explain his condition and has implications for his children. Prior to his next appointment, he flags this issue via his VT so that his rheumatologist can research the genetic variant, provide an opinion on pathogenicity, and give appropriate advice on the need for family genetic testing at the next appointment. This rapid communication of the patient's priorities improves patient-centred care (45).

### Case 4—PGx-guided prescribing of an antidepressant

An adolescent woman with major depressive disorder and social anxiety disorder has responded poorly to good therapeutic trials of two previous selective serotonin reuptake inhibitors (SSRIs). With pharmacogenomic test results in hand (CYP2D6 rapid metaboliser and CYP2C19 normal metaboliser), her treating psychiatrist uses VTHM to calculate her PGxBS (31) and access the latest CPIC® (Clinical Pharmacogenomics Implementation Consortium) guidelines during the prescribing of her next antidepressant (46). Such PGx-guided antidepressant prescribing may lower the risk of intolerable adverse effects and improve the chances of remission in patients with major depressive disorder (47, 48).

### Case 5—model-informed precision dosing of a narrow therapeutic index drug

A new molecularly targeted oral anti-cancer drug (protein kinase inhibitor) with a narrow therapeutic index is to be taken chronically by an octogenarian who has multiple comorbidities and requires polypharmacy e.g., renal, liver and cardiac impairment, and several drugs that are CYP and transporter inhibitors and/or inducers. It is impossible for a drug development program to consider this level of patient complexity and to provide an evidence-based dosing recommendation in the prescribing information to be used post marketing. In this case, the treating physician uses VTHM as a precision dosing tool by updating the patient's VT with covariates known to influence the pharmacokinetics of the new anti-cancer drug (16, 49, 50). Simulations show that half the recommended dose will obtain the target steady-state concentration in the patient, and this dose is commenced with usual clinical monitoring. Similar precision dosing strategies are known to improve the toxicity profiles of many molecularly targeted oral anti-cancer drugs without compromising efficacy (51, 52).

## Trial and error—what if scenarios

As with aeronautical engineering, VT can be used to simulate real scenarios that are difficult or impossible to study during drug development or post-marketing. The pharmacokinetics (±pharmacodynamics) of multiple drugs used together in patients with several comorbidities (such as Case 5) is an example where various drug regimens can be tested *in silico* prior to selecting the best one for the individual patient. Outcomes of non-pharmacological interventions can be incorporated in cases where this is well-understood e.g., the potential effects of a low-calorie ketogenic diet on glucose tolerance and body weight (53). As confidence with the models increases, so will confidence in the outcomes and subsequent recommendations.

## Big data, big decisions

As VTHM becomes more sophisticated, and with increasing coverage of certain patient populations, it is envisaged that the data curated from VTHM could be used for health-economic analyses, which, in turn, could inform healthcare policy (54). There would be extensive data mining to help answer pharmacoepidemiology questions (55). Future generations could extract benefits from these data and establish “lessons learnt” regarding healthcare patterns and barriers to success. For example, artificial intelligence and machine learning would rationalise drug and dose selection from various guidelines together with real-world data on the outcomes from similar patients with regard to ethnic status, socioeconomic status, genetic makeup, medical conditions and co-medications (56, 57). Powerful “Precision Medicine 3.0” algorithms (58) could then select one treatment regimen over another based on novel “fingerprints” of disease and treatment biomarkers, thus making high-level recommendations to policymakers. A patient's VT could indeed “live” on after death, providing valuable data on a human life for future generations.

## Challenges on the path from concept to reality

The functionalities on the spokes of VTHM (Figure 1) are well-established in healthcare to improve clinical outcomes e.g., e-prescribing (12). The VT hub is new in healthcare, but routine in the pharmaceutical industry (59), and there are many scientific, technical, clinical and logistical challenges to overcome prior to acceptance as a CDSS and precision dosing tool in the clinic and at the bedside (16). Perspectives from regulators, payers, clinicians, and patients about such tools are addressed in the previous reviews on MIPD (11, 60). The major challenge of VTHM is to unite the individual components of the system into one smart phone application (Figure 1). This is a formidable undertaking not underestimated by the developers. The technology is disruptive, which understandably brings opposition from those with affected business interests. Therefore, the business model for VTHM is complex and involves partnerships with other e-health system companies and healthcare service providers. The legal and regulatory frameworks for some components of VTHM are evolving and will require novel solutions, but there is already strong regulatory support for precision dosing tools under medical device guidance (11, 60). Issues of data privacy and data security must be addressed (61). Monitoring the clinical utility of VTHM is also critical, particularly when technical changes are implemented e.g., the addition of new drugs to the VT hub. A collaborative effort across the pharmaceutical, regulatory, academic, and healthcare sectors may be required for this large job. Finally, some patients will be unable to use VTHM, such as the young, elderly and the critically unwell. Caregivers would help bridge this gap.

## Conclusions

Escalating healthcare costs and adverse clinical outcomes have not been improved adequately by e-health platforms that are locally oriented with restricted access. In this commentary, a conceptual patient-centred smart phone application called VTHM is proposed that works as a combined personal- and electronic-healthcare record. The platform is revolutionary because it transfers the control of health information from service providers to patients. The overachieving aims of VTHM are simple but ambitious—to lower healthcare costs and improve patient care by decreasing healthcare fragmentation. With big ambitions come big challenges, and those for VTHM are formidable and not underestimated. But the potential benefits of VTHM would be worth the effort. Notably, the VTHM platform is designed to operationalize current and future precision medicine initiatives, such as access to molecular diagnostic results, PGx-guided prescribing, and MIPD.

## Data Availability

The original contributions presented in the study are included in the article/Supplementary Material, further inquiries can be directed to the corresponding author.
